# Photobiomodulation Attenuates Neurotoxic Polarization of Macrophages by Inhibiting the Notch1-HIF-1α/NF-κB Signalling Pathway in Mice With Spinal Cord Injury

**DOI:** 10.3389/fimmu.2022.816952

**Published:** 2022-03-17

**Authors:** Yangguang Ma, Penghui Li, Cheng Ju, Xiaoshuang Zuo, Xin Li, Tan Ding, Zhuowen Liang, Jiawei Zhang, Kun Li, Xuankang Wang, Zhijie Zhu, Zhihao Zhang, Zhiwen Song, Huilin Quan, Xueyu Hu, Zhe Wang

**Affiliations:** ^1^Department of Orthopaedics, Xijing Hospital, Fourth Military Medical University, Xi’an, China; ^2^Department of Orthopaedics, 967 Hospital of People’s Liberation Army Joint Logistic Support Force, Dalian, China

**Keywords:** photobiomodulation, spinal cord injury, inflammatory response, macrophages, polarization, Notch1

## Abstract

Spinal cord injury (SCI) is a catastrophic disease with a complex pathogenesis that includes inflammation, oxidative stress, and glial scar formation. Macrophages are the main mediators of the inflammatory response and are distributed in the epicentre of the SCI. Macrophages have neurotoxic and neuroprotective phenotypes (also known as classically and alternatively activated macrophages or M1 and M2 macrophages) that are associated with pro- or anti- inflammatory gene expression. Our previous study demonstrated that photobiomodulation (PBM) alters the polarization state of macrophages in the SCI region towards the M2 phenotype and promotes the recovery of motor function in rats with SCI. However, the mechanism by which PBM promotes SCI repair remains largely undefined. This study is based on the replacement of conventional percutaneous irradiation with implantable biofibre optic *in vivo* irradiation. The aim was to further investigate the effects of PBM on SCI in mice under new irradiation patterns and its potential mechanisms of action. PBM was administered to male mice with clamped SCI for four consecutive weeks and significantly promoted the recovery of motor function in mice. Analysis of the macrophage phenotypes in the epicentre of the SCI in mice showed that PBM mainly inhibited the neurotoxic activation of macrophages in the SCI area and reduced the secretion of inflammatory factors such as IL-1α and IL-6; PBM had no effect on M2 macrophages. Immediately afterwards, we constructed *in vitro* models of the inflammatory polarization of macrophages and PBM intervention. We found that PBM attenuated the neurotoxicity of M1 macrophages on VSC 4.1 motor neurons and dorsal root ganglion (DRG) neurons. The effects of PBM on neurotoxic macrophages and the possible mechanisms of action were analysed using RNA sequencing (RNA-seq), which confirmed that the main role of PBM was to modulate the inflammatory response and immune system processes. Analysis of the differentially expressed genes (DEGs) associated with the inflammatory response showed that PBM had the most significant regulatory effects on genes such as interleukin (IL)-1α, IL-6, cyclooxygenase-2 (COX-2), and inducible nitric oxide synthase (iNOS) and had obvious inhibitory effects on inflammation-related Notch1 and hypoxia-inducible factor-1α (HIF-1α) pathway genes. RNA-seq analysis of the effect of PBM on gene expression in resting-state macrophages and M2 macrophages did not show significant differences (data not shown). In conclusion, PBM promoted better motor recovery after SCI in mice by inhibiting the neurotoxic polarization of macrophages and the release of inflammatory mediators by acting on the Notch1-HIF-1α/NF-κB Signalling Pathway.

## Introduction

Spinal cord injury (SCI) is a central nervous system injury caused by physical factors, such as car accidents, violence, and fall injuries, that often leads to autonomic, sensory, and motor nerve dysfunction below the plane of injury; it is often accompanied by paralysis and lifelong disability in severe cases and may also lead to mental and psychological disorders, thus placing a heavy burden on patients, their families, and society (1–3). Previous studies have found that spinal cord injuries can be divided into two stages according to pathological development: primary and secondary injuries (3, 4). Primary injury is irreversible and refers to the direct effect of trauma, including compression, contusion, and shear forces, to the spinal cord. Secondary injury is a continuation of the primary injury; its pathological process mainly includes the inflammatory response, oxygen-free radical formation, local ischaemia, and apoptotic necrosis, which can last for weeks or months and lead to a gradual expansion of the lesion area and further aggravation of nerve damage (5). In particular, the secondary inflammatory response is considered to be a key in the prognosis of SCI (6, 7).

Macrophages are the most important immune cells in the secondary inflammatory response after SCI, and their polarization status plays a decisive role in the local microenvironment of the injured area (8, 9). Macrophages are recruited to the injury epicentre 2-3 days after SCI; macrophage-mediated inflammation peaks approximately 7 days after injury and plays an important role in secondary SCI (10, 11). In response to different microenvironmental signals in the injury zone, macrophages can exhibit M1 (proinflammatory; classically activated) or M2 (anti-inflammatory; alternatively activated) polarization states; M1 macrophages are neurotoxic and M2 macrophages promote regeneration of damaged nerves (12). M1 macrophages express high levels of iNOS and proinflammatory cytokines (e.g., IL-6, IL-1α, and IL-1β), which exert cytotoxic effects on neurons and glial cells (6). In contrast, M2 macrophages produce high levels of arginase 1 (Arg1), anti-inflammatory factors (e.g., IL-10 and TGF-β), and neurotrophic factors (e.g., BDNF and NGF), which exert immunomodulatory, tissue repair, and functional remodelling effects (8). In a mouse model of SCI, the macrophages are mainly proinflammatory (12, 13). Inhibition of proinflammatory M1 activation or suppression of M1 inflammatory mediator expression may be a promising approach for the treatment of SCI. PBM, also known as low-level laser therapy (LLLT), is a classic physical therapy method that has significant anti-inflammatory and tissue repair effects (14). Relevant studies have shown that PBM can change the polarization state of macrophages in the SCI area of rats to the M2 phenotype, reduce the apoptosis of neurons and oligodendrocytes, promote axonal regeneration, and ultimately promote the recovery of neurological function (15, 16). However, the mechanisms by which PBM regulates macrophage polarization and promotes SCI repair remain unclear.

Notch signalling, a highly conserved signalling pathway, is involved in multiple biological processes, such as the determination of cell fate, differentiation, and apoptosis (17, 18). Mammals express four Notch receptors (Notch-1 to -4) and five Notch ligands (Delta-like-1, -3, and -4, and Jagged-1 and -2). The binding of the ligand to the receptor results in the shedding of the Notch extracellular domain, followed by the release of the Notch intracellular domain (NICD) by γ-secretase. The NICD is translocated to the nucleus, where it binds to the transcription factor RBP-J, which activates Notch downstream target genes, mainly hairy and enhancer of split (Hes) isoforms 1 and 5. The NICD also interacts with HIF-1α (19, 20), a major regulator of glycolysis, which is associated with M1 activation (21, 22). Additionally, in LPS-induced activation of macrophages, NICD1 is recruited to mitochondrial DNA (mtDNA), and the respiratory chain components encoded by mtDNA are upregulated in a Notch-dependent manner (20). Taken together, these findings suggest that Notch plays an important role in linking macrophage metabolism and M1 activation. Furthermore, γ-secretase inhibitors (DAPT) could exert inhibitory effects on Notch to attenuate LPS-stimulated M1 gene expression (23).

We conducted the experiments described below to study the regulatory effect of PBM on macrophages in the SCI area of mice and their potential molecular mechanisms. In this study, mouse bone marrow-derived macrophages (BMDMs) were extracted, and models of macrophage inflammatory polarization and PBM treatment were constructed *in vitro*. Transcriptome analysis and validation of differentially expressed genes in classically activated macrophages under PBM were performed. We found that PBM attenuated the neurotoxic polarization of macrophages and the secretion of inflammatory factors by inhibiting the Notch1-HIF-1α/NF-κB signalling pathway. Our findings provide novel insight into elucidating the molecular mechanisms of the anti-inflammatory and tissue repair effects of PBM. We suggest that Notch1 and HIF-1α may serve as new therapeutic targets for SCI.

## Materials and Methods

### Animals and Ethics Statement

Six- to eight-week-old male C57BL/6 mice, each weighing approximately 18-20 g, were provided by the Animal Experiment Centre of the Fourth Military Medical University (Xi’an, Shaanxi, China). Animals were housed in clean and warm cages at an ambient temperature of 22-25°C on a 12-hour light/dark cycle with an adequate supply of food and water. The animal protocol was reviewed and approved by the Ethics Committee of the Fourth Military Medical University. All operations were performed to minimize the number of animals used and to reduce animal suffering.

### Spinal Cord Injury Model

Male C57BL/6 mice, 8-10 weeks old, were used to generate the SCI models. Based on our previous study (15, 24), a standardized bilateral spinal cord compression injury model in mice under aseptic conditions was constructed. Mice were anaesthetized by intraperitoneal injection of 0.6% sodium pentobarbital (10 mL/kg). After disinfection, the skin, subcutaneous tissue, and paravertebral muscles were separated layer by layer, and the T9 vertebral body was positioned under the microscope. Then, the T9 spinous process and vertebral plate were removed with fibre bite forceps to fully expose the spinal cord without injuring it. Dumont tethered forceps (Fine Scientific Tools, Heidelberg, Germany) were modified by adding a metal spacer between the blades to achieve a 0.3-mm gap at full closure. The modified forceps were fully inserted around both sides of the spinal cord in a vertical direction, and the spinal cord was clamped with the forceps maximally closed for 30 s. Both lower limbs of the mice were stretched, and the tails were twitched. After clamping, absorbable sutures were used to moderately fix the front end of the medical diffusing optical fibre (Xi’an Laser Tech Medical Technology Co., Ltd., Shaanxi, China) to the spine and soft tissues of the 8th thoracic segment of the mice (25). The muscle layer and skin were sutured together, and the skin was disinfected with iodophor. Mice with only exposed spinal cord tissue and no clamp injury were used as the sham-operated group. After the operation, each mouse was placed individually in a warm box until they woke up. The mice were given artificial bladder massages twice a day to prevent urinary tract infection. If the mice developed lower limb oedema or wound infection, they were excluded from the experiment.

### Photobiomodulation Therapy

Mice treated with SCI and sham surgeries were randomly divided into the PBM therapy group and the blank control group, each containing 40 mice. Mice in the PBM therapy group were anaesthetized by intraperitoneal injection of 0.6% sodium pentobarbital (10 mL/kg) and placed in a dark cage at an ambient temperature of 25°C. A continuous 808-nm semiconductor laser (MW-GX-808, Lei Shi Optoelectronics Co., Ltd. Changchun, China) and its matched medical diffusing optical fibre were used for PBM therapy in mice. The safety of the optical fibre and the irradiation parameters has been validated in piglet models (25, 26). The highly transparent medical silica coating on the fibre surface ensures flexibility and biocompatibility without affecting its optical properties. The optical fibre is cylindrical with a diameter of 600 μm. The output power of the fibre was tested using a calibrated optical sensor to confirm that the output power of the fibre was consistent with the set power. We irradiated the SCI area of mice with a power density of 50 mW/cm^2^ for 50 minutes once a day.

### Behavioural Analysis

The recovery of motor function in the hindlimbs of mice was assessed by the Basso Mouse Scale (BMS) (27). Each mouse was pretrained and placed individually in an open field. The method measures hindlimb joint movement, trunk position and stability, forelimb and hindlimb coordination, paw position, toe clearance, and tail position. All mice were observed by two independent investigators blinded to the treatment groups. The scores were measured before surgery and at 1, 3, 7, 14, 21, and 28 days postinjury (dpi). Scores from 0 to 9 represent total paralysis to normal movement, respectively. The Louisville Swimming Scale (LSS) was performed to assess the recovery of motor function after SCI (28). Mice were placed in a water-filled tank and trained to swim from one side to the other. Forelimb dependence, hindlimb movement and alternation, body angle, and trunk stability were recorded and assessed according to the LSS. Each mouse was tested twice, and the average of the two tests was taken as the final score.

### Tissue Processing

Mice were anaesthetized and perfused with 4% paraformaldehyde for cardiac perfusion, and 2-cm-long spinal cord segments that were centred on the injury site were dissected, incubated in the same fixative for 6 hours, transferred to 25% sucrose solution for dehydration and incubated at 4°C until the tissue sank. The tissue was then placed face-up on the specimen tray and encapsulated in OCT embedding agent, and the specimen tray was placed on the quick-freezing table of a cryostat (CM1900, Leica) for rapid freeze embedding. When the OCT had frozen, tissue sectioning commenced. The sagittal direction of the spinal cord was extended, and consecutive sections of approximately 12-µm thickness were obtained, followed by fixation of the sections on slides. Sections of the area near the epicentre were selected for each subject, stored at -20°C, and prepared for immunofluorescence. After the mice were anaesthetized, the hearts were rinsed with saline, and approximately 2-cm-long spinal cord segments were collected from the injury site, rapidly frozen in liquid nitrogen, and stored at -80°C. The prepared samples were used for western blot analysis.

### Western Blot Analysis

RIPA lysis buffer supplemented with protease and phosphatase inhibitor cocktail (Thermo Fisher) was used to extract proteins from the spinal cord tissue and cultured cells. The protein concentration was determined by the BCA method. Equal amounts of protein (30-50 µg) were isolated from each set of samples, separated by SDS–PAGE (10%), and transferred to NC membranes (EMD Millipore Corp). Membranes were blocked with 5% skimmed milk powder for 1 hour at room temperature and incubated overnight at 4°C with the following specific primary antibodies: iNOS (13120S, Cell Signaling Technology, 1:1000), Arg-1 (93668S, Cell Signaling Technology, 1:1000), β-Actin (3700S, Cell Signaling Technology, 1:1000), Notch1 (3608S, Cell Signaling Technology, 1:1000), HIF-1α (bs-0737R, Bioss, 1:1000), NF-κB (8242S, Cell Signaling Technology, 1:1000), and p-NF-κB (3033S, Cell Signaling Technology, 1:1000). After washing with TBST, the membranes were incubated with the corresponding secondary antibodies for 1 hour. The bands were developed with western blotting-enhanced chemiluminescent solution (Millipore), and the images were captured with an Amersham Imager 600 Station (GE Healthcare, Stockholm, Sweden). The greyscale value of each band was measured using ImageJ software.

### Immunofluorescence

Frozen sections of spinal cord tissue or cultured cells were washed three times with phosphate-buffered saline (PBS) for 5 minutes each and then incubated with 1% donkey serum containing 0.3% Triton X-100 for 30 minutes. The sections or cells were incubated with primary antibody overnight at 4°C. The following primary antibodies were used: anti-F4/80 (ab6640, Abcam, 1:150), anti-iNOS (ab49999, Abcam, 1:200), anti-Arg1 (93668S, Cell Signaling Technology, 1:50), anti-CD86 (ab119857, Abcam, 1:150), anti-mannose receptor (CD206) (ab64693, Abcam, 1:200), anti-Notch1 (4380T, Cell Signaling Technology, 1:200), and anti-β-III-tubulin (ab78078, Abcam, 1:300). Incubation with the corresponding secondary antibody was then performed for 2 hours at 37°C. Finally, the nuclei were stained with DAPI. Fluorescence images were acquired by fluorescence microscopy (BX51, Olympus), and the fluorescence intensity was analysed by ImageJ software.

### Extraction and Treatment of Mouse BMDMs

Mouse BMDMs were extracted in the manner we have previously reported (29). Briefly, the femur and tibia of 6- to 8-week-old C57BL/6 mice were extracted, and their bone marrow cavities were flushed with PBS. The flushed cell suspension was digested with erythrocyte lysis solution and centrifuged at 300 × g for 5 minutes. The supernatant was discarded. The cell-containing pellet was resuspended in pretreated Dulbecco’s modified Eagle’s medium (DMEM) containing 10% FBS, 1% penicillin/streptomycin solution, and 10 ng/mL macrophage colony-stimulating factor (M-CSF; R&D Systems). Bone marrow cells were counted using a haemocytometer. The concentration was adjusted to 2×10^6^ cells/mL in DMEM. Two millilitres of DMEM containing primary bone marrow macrophages was added to each well of a 6-well plate. BMDMs were induced to differentiate into M0 macrophages with M-CSF-conditioned medium (10% FBS, 1% penicillin–streptomycin double-antibody, DMEM high-glucose medium, 10 ng/mL M-CSF). Cells were differentiated in fresh conditioned medium for 7 days until the cells matured in culture.

Mature M0 macrophages were then polarized to M1 macrophages after the addition of 100 ng/mL lipopolysaccharide (LPS; Sigma–Aldrich) and 20 ng/mL interferon-γ (IFN-γ; Sigma–Aldrich) to the medium. For the PBM intervention model of M1 macrophages, we placed mouse BMDMs costimulated with LPS and IFN-γ in a sterile dark box at a constant temperature and irradiated the cells in a vertical direction using a semiconductor laser equipped with a fibre uniform lens (COL-12K, Lei Shi Optoelectronics Co., Ltd. Changchun, China). PBM intervention was administered while LPS plus IFN-γ were added to BMDMs to induce neurotoxic M1 macrophage activation. PBM treatment with an irradiation power of 6 mW/cm^2^ and an irradiation duration of 7 minutes was performed every 12 hours. Cells in the control group were also placed in the dark chamber, but no PBM therapy was performed. **Supplementary Tables 1** and **2** provide information on the semiconductor laser equipment and the specific parameters of the PBM therapy.

### Flow Cytometry

BMDMs were cultured *in vitro* with M-CSF-conditioned medium. After culturing for 7 days, BMDMs were stimulated for 48 hours with PBS and 100 ng/mL LPS plus 20 ng/mL IFN‐γ to polarize them into M0 and M1 macrophages, respectively. After cell maturation, 0.25% trypsin with 0.02% EDTA (Gibco, Grand Island, USA) was added, and 6‐well plates were plated into incubators for 2 minutes during digestion. Then, DMEM with 10% FBS was added to terminate digestion, the cells were placed into a centrifuge at 300 × g for 5 minutes, and the supernatant was discarded. Cells were resuspended in flow cytometry liquid and blocked with 20 μL of rat serum. Next, 40 μL of the primary antibody mix (BV510-CD11b, 1:200, BioLegend, 101245; APC-F4/80, 1:50, eBioscience, 17-4801-82; PE-CD86, 1:200, BioLegend, 105014) was added and the cells were incubated for 15 minutes on ice in the dark. Then, the cells were washed twice with flow cytometry liquid and assessed on a flow cytometry machine (Beckman Coulter, CA, USA).

### RT–PCR

An OMEGA RNA extraction kit (BioLegend, San Diego, CA) was used according to the manufacturer’s protocol to extract the total RNA. The RNA obtained was reverse transcribed using PrimeScript RT Master Mix (TaKaRa Bio Inc, Kusatsu, Japan) according to the manufacturer’s protocol. The beta‐actin housekeeping gene was used for the normalization of gene expression by parallel amplification. The relative expression levels of the target mRNAs were calculated. The RT–PCR data were analysed by the 2^−ΔΔCT^ method. The PCR primer sequences are listed in **Supplementary Table 3**.

### Neuronal Culture and Treatments

Ventral spinal cord 4.1 (VSC 4.1) motoneurons were grown in a fully humidified incubator at 37°C with 5% CO_2_ in DMEM containing 10% FBS and 1% penicillin/streptomycin. Dorsal root ganglia (DRGs) were extracted from Sprague–Dawley rat neonates (P0–P2) using the method we reported previously (30). The DRGs were completely cut up and digested with trypsin solution (0.125%) and type IV collagenase solution (0.1%) for 30 minutes. The digested DRGs were rinsed well, and digestion was stopped in DMEM-F12 supplemented with 20% FBS. Then, the cells were centrifuged at 1,000 rpm for 5 minutes. The DRG neurons were subsequently suspended in neurobasal cultures with the addition of B27 and 1% penicillin/streptomycin solution. The DRG neurons were grown in 6-well plates at a density of 10,000 cells per well.

To investigate the effect of PBM on the neurotoxicity of M1 macrophages. We collected the culture supernatant of macrophages after 24 hours of LPS plus IFN-γ treatment and labelled it as M1 macrophage-conditioned medium (M1CM). The conditioned medium was filtered with a 0.22-μm membrane to remove cellular residue. At the same time, the M1CM was treated with PBM (M1CM + PBM), and the conditioned medium of resting-state macrophages (M0CM) was collected. Half of the VSC 4.1 motor neurons and DRG neuronal cultures were replaced with macrophage-conditioned medium (MCM). Neurons were cultured in mixed medium for 24 hours to assess the effect of macrophages on neurons.

### Annexin V-FITC/PI Assay for Apoptosis

To measure VSC 4.1 motoneuron apoptosis, neuronal cells treated with three different sets of MCM for 24 hours were collected using the Alexa Fluor 488-Annexin V Dead Cell Apoptosis Kit (Thermo Fisher) according to the manufacturer’s protocol and double-stained with propidium iodide (PI) and Alexa Fluor 488-Annexin V. The stained cells were analysed by flow cytometry (Beckman F500). A minimum of 10,000 cells were analysed in each sample.

### Quantification of Neurite Outgrowth

For the neurite outgrowth assays, the extent of neurite outgrowth under different experimental conditions was compared by measuring the mean neurite length of each neuron. The NeuronJ plug-in of the image analysis software suite NIH ImageJ was used to measure neurite length. Each experimental condition was repeated in three wells. Four images with 200× magnification were randomly obtained in each well. The neurites of all neurons in each image were tracked, and the number of DRG neurons in each image was counted. There were 5 neurons in each image; therefore, 20 neurons were measured in each well. The total neurite length of the 20 neurons was divided by the number of neurons to calculate the average neurite length of neurons in each well. The average value of 3 wells under each culture condition was taken, and the average value of 3 independent cultures was used to obtain the average group value of the neurite length of each neuron.

### RNA-seq and GO Analysis

Twelve hours after PBM intervention, total RNA was collected from M1 macrophages in the PBM intervention group and the blank control group using a TRIzol kit (Takara). RNA quality was examined by gel electrophoresis and with a Nanodrop spectrophotometer (Thermo, Waltham, MA, USA). Enrichment of mRNA, fragmentation, reverse transcription, library construction, and HiSeq X Ten were performed by Genergy Biotechnology Co. Ltd. (Shanghai, China). Cufflinks methods were used for the determination of expression values (31). GO analysis was carried out with clusterProfiler software. P ≤ 0.05 was considered to indicate significance.

### ELISA

The levels of IL-1α, IL-6, and COX-2 were measured in macrophage cultures from the M0, M1 and M1+PBM groups at 24 and 48 hours after PBM intervention. An enzyme-linked immunosorbent assay (ELISA) kit (Meimian Industrial) was used according to the manufacturer’s instructions. The levels of IL-1α and IL-6 in the spinal cord tissues of the sham-operated, 7 dpi, and 7 dpi + PBM groups were assessed with ELISA kits (Meimian Industrial). The injured spinal cord tissue was homogenized in PBS, and the tissue homogenization supernatant was collected, manipulated and analysed according to the instructions provided by the reagent vendor.

### Treatment of Cells With Inhibitors

To target Notch signalling in M1 macrophages, the γ-secretase inhibitor DAPT (S2215, Selleckchem) was used to treat LPS- and IFN-γ-treated macrophages. Macrophages were pretreated with two concentrations of DAPT (20 μM and 40 μM), before the addition of LPS and IFN-γ for 1 hour. An equal volume of DMSO was added as a vector control for DAPT.

### Virus Construction and Transfection

For adenovirus infection (Hanbio Biotechnology, Shanghai, China), cells were seeded in 6-well plates at a density of 1 × 10^6^ cells per well. After maturation of macrophages, the cells were infected with adenoviruses expressing GFP (Ad-GFP) or intracellular domain of Notch1 (NICD1) (Ad-NICD1-GFP) with a multiplicity of infection (MOI) of 100. All transfections were performed with the help of polybrene according to the manufacturer’s instructions. Forty-eight hours after transfection, M1 macrophages were harvested for immunoblotting assays.

### Statistical Analysis

All the data from at least three independent experiments in the present study were first processed by GraphPad Prism version 8.3.0 software and are presented as the mean ± standard deviation (SD). Means were compared using Student’s t test where applicable. Differences among more than two groups were statistically analysed using one-way analysis of variance (ANOVA) followed by *post hoc* Tukey’s multiple comparisons test. P< 0.05 was taken to indicate a statistically significant difference; subsequently p values are indicated with asterisks (*P< 0.05; **P< 0.01; and ***P< 0.001).

## Results

### PBM Promotes the Recovery of Motor Function in SCI Mice and Reduces the Activation of Neurotoxic Macrophages in the Epicentre of the Injury

**Figure 1:**
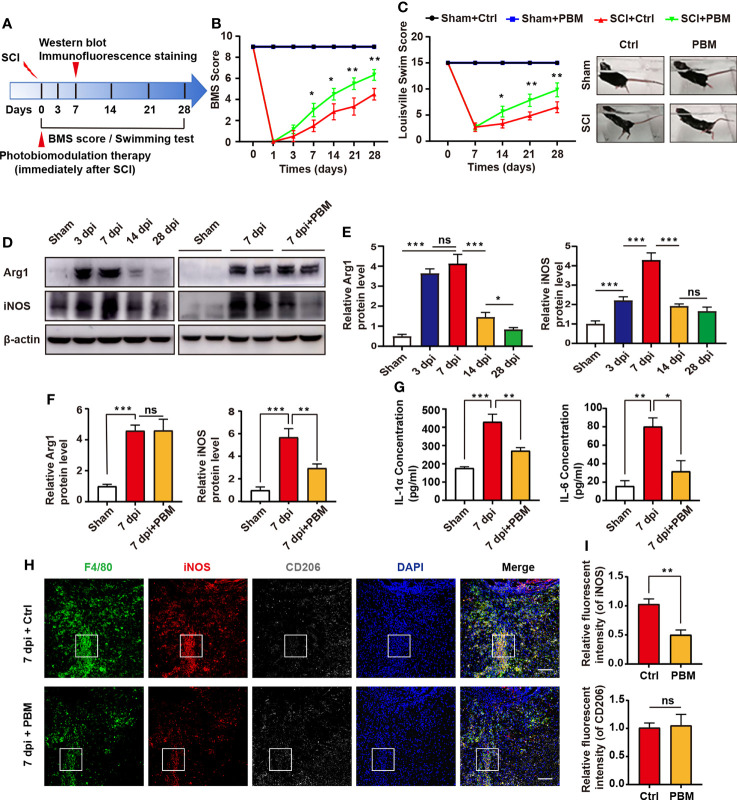
PBM promotes the recovery of motor function in mice with compressive SCI and reduces the neurotoxic polarization of macrophages in the injury area. **(A)** Experimental paradigm. **(B)** Statistical analysis of the Basso Mouse Scale (BMS) score in the sham surgery and SCI groups over a period of 28 days (n = 6 mice per group at each time point). **(C)** Photographs of varying degrees of trunk instability observed after SCI or sham surgery and statistical analysis of the Louisville Swimming Scale (LSS) over 28 days in the control and PBM groups (n = 6 mice per group at each time point). **(D–F)** Immunoblotting of Arg1 (M2 macrophage marker) and iNOS (M1 macrophage marker) at 3 dpi, 7 dpi, 14 dpi, and 28 dpi or after sham surgery (28 days after the sham operation) (n = 3 mice per group at each time point). **(G)** ELISA results for IL-1α and IL-6 in the injured spinal cords of each group at 7 dpi. **(H, I)** Representative immunohistochemical staining of F4/80 (macrophage marker), iNOS (M1 macrophage marker) and CD206 (M2 macrophage marker) in the epicentre of the injured spinal cord of the control and PBM groups at 7 dpi (scale bar = 100 μm). *P < 0.05, **P < 0.01, ***P < 0.001; ns, not significant.

**Figure 1A** is the experimental design in this study. The BMS and LSS score were used to evaluate the motor function of mice in different treatment groups. As shown in **Figures 1B, C**, in the sham operation group, PBM did not affect the swimming test or BMS score of the mice. These results suggest that PBM does not injure the spinal cord tissue of mice. The BMS score of mice with SCI were compared with the control group and the PBM group. We found that at 7 dpi, the BMS score of the PBM group was higher than that of the control group, and this difference was maintained until the 28 dpi. The recovery of motor function in the PBM group was significantly better than that in the control group (**Figure 1B**). The LSS score of SCI mice also showed that the recovery of lower limb motor function in the PBM group was significantly better than that in the blank control group (**Figure 1C**). Previous reports have shown that macrophages are recruited to the epicentre of injury after SCI and play an important role in secondary inflammation (1, 8, 11). To investigate the characteristics of macrophage-associated inflammatory responses in the SCI area with the time of injury, we extracted the spinal cord tissue of mice in the sham operation group on the 28th day after the operation and the spinal cord tissue of the mice in the SCI group at 3 dpi, 7 dpi, 14 dpi, and 28 dpi. The protein levels of iNOS (M1 marker) and Arg1 (M2 marker) in each group were detected by western blotting. The results showed that the expression levels of macrophage polarization-related iNOS and Arg-1 peaked at approximately 7 dpi and then decreased gradually (**Figures 1D, E**). At 7 dpi, PBM decreased the expression of iNOS in the injured area at the protein level but did not affect the expression of Arg1 (**Figures 1D, F**). At the same time, we detected the expression of neurotoxicity-related cytokines. The expression of the cytokines IL-1α and IL-6 was elevated after SCI, and PBM reduced the expression of these neurotoxic cytokines. This may be a potential mechanism by which PBM inhibits neurotoxic polarization of macrophages and promotes recovery of motor function in mice (**Figure 1G**). Subsequently, we examined the activation levels of macrophages in the 7 dpi + Ctrl and 7 dpi + PBM groups. F4/80^+^iNOS^+^ cells were considered M1 macrophages, and F4/80^+^CD206^+^ cells were considered M2 macrophages. M1 macrophage activation was inhibited in the 7 dpi + PBM group compared with the 7 dpi + Ctrl group, whereas M2 macrophage activation did not differ between the two groups (**Figures 1H, I**). Previous studies have confirmed that macrophages of peripheral origin can stay at the site of SCI for a long period and occupy the epicentre of the injury (8). Next, we isolated mouse BMDMs and induced their polarization to the M1 phenotype *in vitro* to further explore the effects of PBM on neurotoxic macrophages and their potential mechanisms of action.

### Identification of BMDMs and Induction of Neurotoxic Macrophages

**Figure 2:**
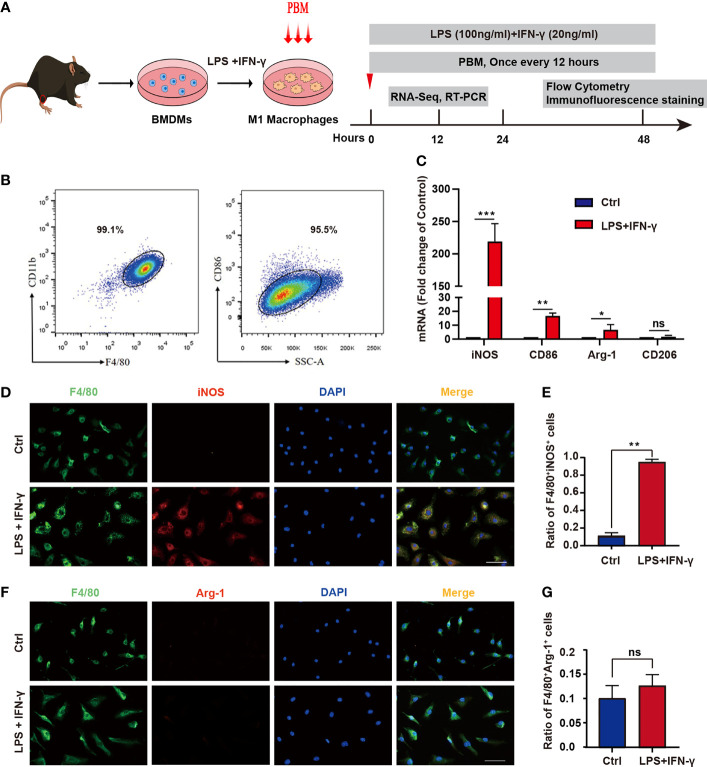
Identification of BMDMs and induction of neurotoxic macrophages. **(A)** Schematic diagram of extraction of mouse BMDMs and induction of M1-type polarization and timeline of the experimental design. **(B)** BMDMs were identified by examining the macrophage markers F4/80 and CD11b using flow cytometry. **(C)** The expression of M1 markers (iNOS and CD86) and M2 markers (Arg1 and CD206) was detected by RT–PCR in the control group and the LPS + IFN-γ-treated group. **(D)** Immunofluorescence staining was used. Red: the M1 macrophage marker iNOS; green: the macrophage marker F4/80; blue: DAPI (nucleated cells) in untreated and LPS + IFN-γ-treated cells. Scale bar = 50 mm. **(E)** The ratio of F4/80^+^iNOS^+^ cells in the control group and in the LPS- and IFN-γ-treated groups. **(F)** Immunofluorescence staining was used. Red: the M2 macrophage marker Arg1; green: the macrophage marker F4/80; blue: DAPI (nucleated cells) in untreated and LPS + IFN-γ-treated cells. Scale bar = 50 mm. **(G)** The ratio of F4/80^+^Arg1^+^ in the control group and in the LPS- and IFN-γ-treated groups (all experiments in this group were independently repeated three times). *P < 0.05, **P < 0.01, ***P < 0.001; ns, not significant.

To further validate the involvement of PBM in the regulation of macrophage polarization, we isolated BMDMs and identified them by detecting the macrophage markers F4/80 and CD11b by flow cytometry (**Figures 2A, B**). The results showed 99.1% double positivity for F4/80 and CD11b, indicating that we successfully cultured M0 macrophages (**Figure 2B**). M0 macrophages were treated with LPS plus IFN-γ, and the percentage of CD86^+^ cells was detected by flow cytometry after 48 hours (**Figure 2A**). The results showed that the percentage of CD86^+^ M1 macrophages reached 95.5% (**Figure 2B**). In addition, the RT–PCR results (**Figure 2C**) showed that the levels of iNOS and CD86 were significantly higher in LPS plus IFN-γ-treated macrophages than in M0 macrophages, indicating that we successfully induced neurotoxic M1 macrophages. Immunofluorescence staining analysis showed that the proportion of F4/80^+^iNOS^+^ cells in LPS plus IFN-γ-treated macrophages was remarkably higher than that in the control group (**Figures 2D, E**). The proportion of F4/80^+^Arg1^+^ cells was low in both the control and the LPS plus IFN-γ group, and there was no statistical difference in the proportion of double-positive cells between the two groups (**Figures 2F, G**). These results suggest that we successfully extracted mouse BMDMs and induced them to differentiate into neurotoxic M1 macrophages with high expression of CD86 and iNOS.

### Photobiomodulation Attenuates the Neurotoxic Effects of M1 Macrophages on Neurons

**Figure 3:**
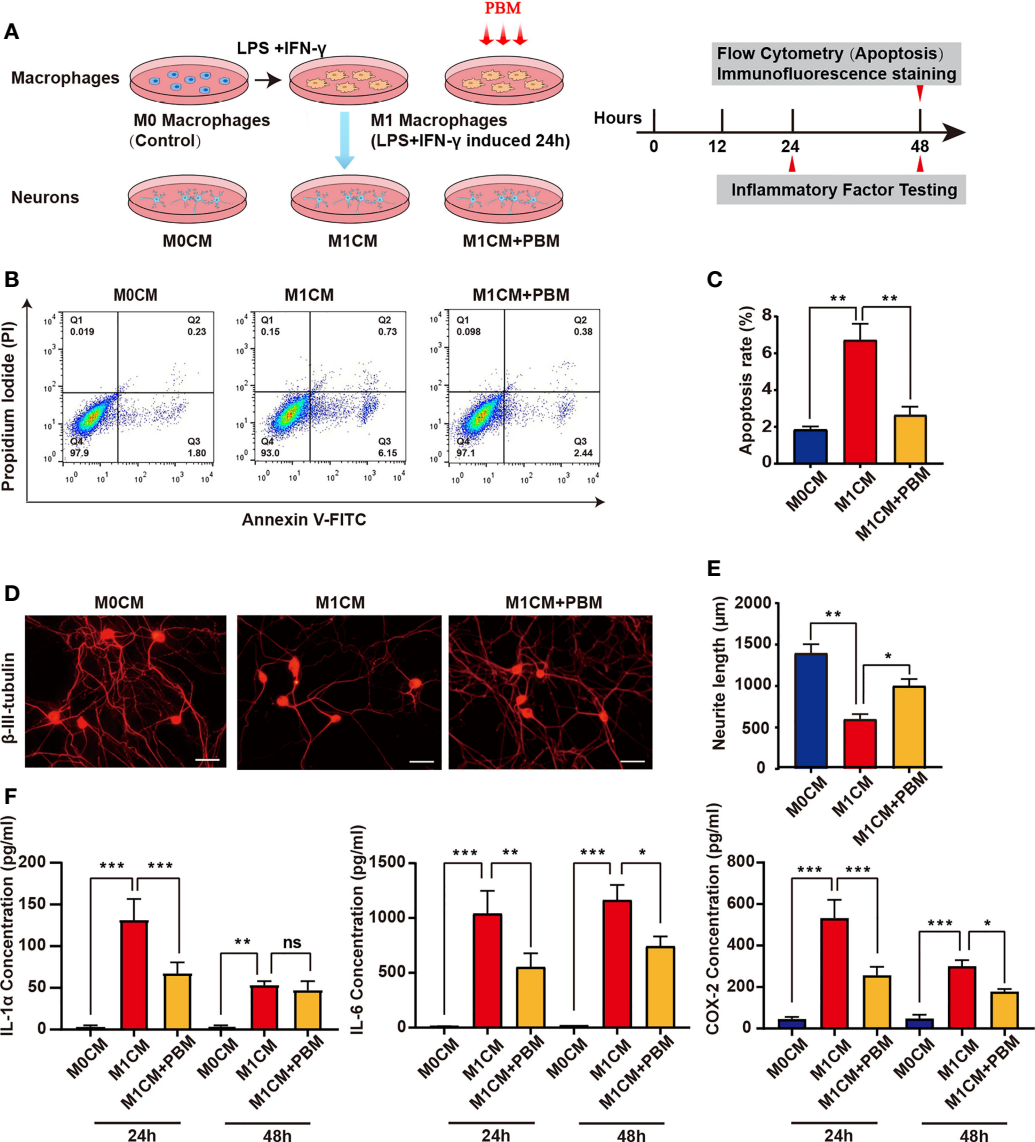
PBM attenuates the neurotoxic effects of M1 macrophages on neurons. **(A)** Schematic diagram of the experiments with macrophages and neurons in pipette coculture. Timeline of the experiments conducted. **(B)** VSC 4.1 motor neurons were treated with M1 macrophage-conditioned medium (M1CM) for 24 hours, followed by double staining with Annexin V-FITC/PI and flow cytometry to detect apoptosis rates. Quadrant 3 (Q3) represents cells stained mainly by Annexin-V (early apoptotic cells), and Q2 represents cells stained by both PI and Annexin-V (late apoptotic). Q1 represents cells stained mainly by PI, and viable cells negative for both Annexin-V and PI appear in Q4. **(C)** The apoptosis rate of neuronal cells is shown in the bar graph. **(D)** Representative images of β-III-tubulin^+^ cultured DRG neurons (n=3 independent experiments for neurite outgrowth assays, scale bars=50 μm). **(E)** A quantification graph of the mean neurite length per neuron in each group. **(F)** ELISA was used to detect the concentration of inflammation-associated cytokines in macrophage-conditioned culture media (all experiments in this group were independently repeated three times). *P < 0.05, **P < 0.01, ***P < 0.001; ns, not significant.

Previous related studies have shown that M1 macrophages induce neuronal death through the release of toxic cytokines (12). To explore the effect of PBM on the neurotoxicity of M1 macrophages, we added MCM to cultured VSC 4.1 motoneurons and DRG neurons and statistically analysed the apoptosis rate of VSC 4.1 motoneurons and the mean neurite length of DRG neurons to assess the survival of neurons (**Figure 3A**). When M1CM was added to VSC 4.1 motor neurons, the apoptosis rate of neurons increased obviously, while PBM reduced the apoptosis of motor neurons induced by M1CM (**Figures 3B, C**). Moreover, we found that M1CM caused axonal retraction in DRG neurons, while PBM reversed the neurotoxic effects of M1CM to some extent (**Figures 3D, E**). To further study whether PBM affects neuronal survival by regulating the secretion of neurotoxic cytokines from macrophages, we evaluated the expression of the common neurotoxic cytokines IL-1α, IL-6, and COX-2 in M0- and M1-conditioned media. PBM had a inhibitory effect on the expression of IL-1α, IL-6 and COX-2 in M1CM. The effect of PBM was time-dependent, with the most pronounced effect at 24 hours; the effect diminished 48 hours after PBM intervention (**Figure 3F**).

### PBM Alters the Transcriptome of M1 Macrophages

**Figure 4:**
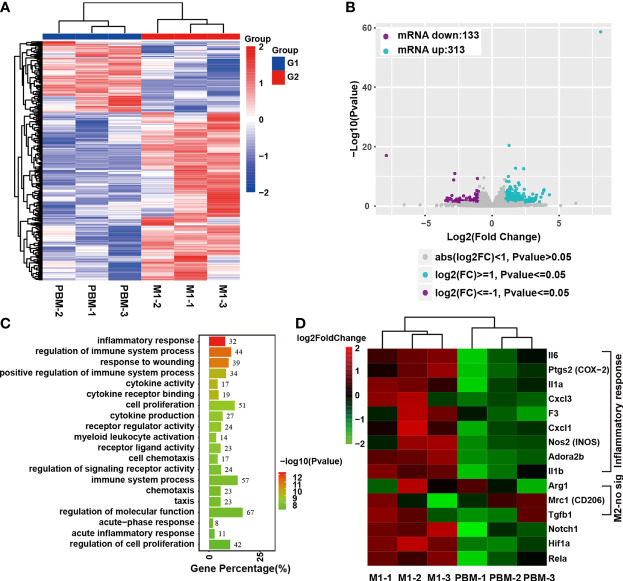
Transcriptome analysis of the effect of PBM on M1 macrophages. **(A, B)** There were 446 genes that were significantly altered, with 313 upregulated and 133 downregulated. **(C)** GO analysis of the differentially expressed genes (DEGs). **(D)** Heatmap of DEGs associated with inflammatory responses in macrophages. The heatmap shows that Notch1 and HIF-1α are downregulated genes related to inflammation regulation. IL-6, PTGS2 (COX-2), and IL-1α were the three genes with the largest fold differences among the inflammatory response-related DEGs, with p values less than 0.01 (this experiment was independently repeated three times).

The effect of PBM on neurotoxic M1 macrophages was studied by RNA-seq. The cluster heatmap of the differentially expressed genes (DEGs) between the sample groups (P ≤ 0.05 and |log2foldchange|≥1) is shown in **Figure 4A**. Scatter plots comparing the PBM treatment group and the control samples are shown in **Figure 4B**. Compared with expression in the blank control group, 446 DEGs were found in M1 macrophages 12 hours after PBM treatment, of which 313 were upregulated and 133 were downregulated (**Figures 4A, B**). In the GO analysis, DEGs were mostly involved in the inflammatory response, regulation of immune system, response to wounding, cytokine activity, etc. (**Figure 4C**). The heatmap of DEGs showed that inflammation-related genes, including IL-6, COX-2, IL-1α, Cxcl3, F3, Cxcl1, and Nos2, were significantly different between the PBM treatment group and the control group, which confirmed the inhibitory effect of PBM on M1 macrophage-related inflammation (**Figure 4D**). Moreover, PBM inhibited macrophage inflammation regulation and oxidation of respiratory chain- and energy metabolism-related signalling pathway genes, such as Notch1 and HIF-1α (**Figure 4D**). In contrast, there was no effect on the expression of M2 macrophage-associated anti-inflammatory genes (e.g., Arg1 and CD206) (**Figure 4D**).

### *In Vitro* Validation of the Regulation of Inflammation-Related Genes by PBM

**Figure 5:**
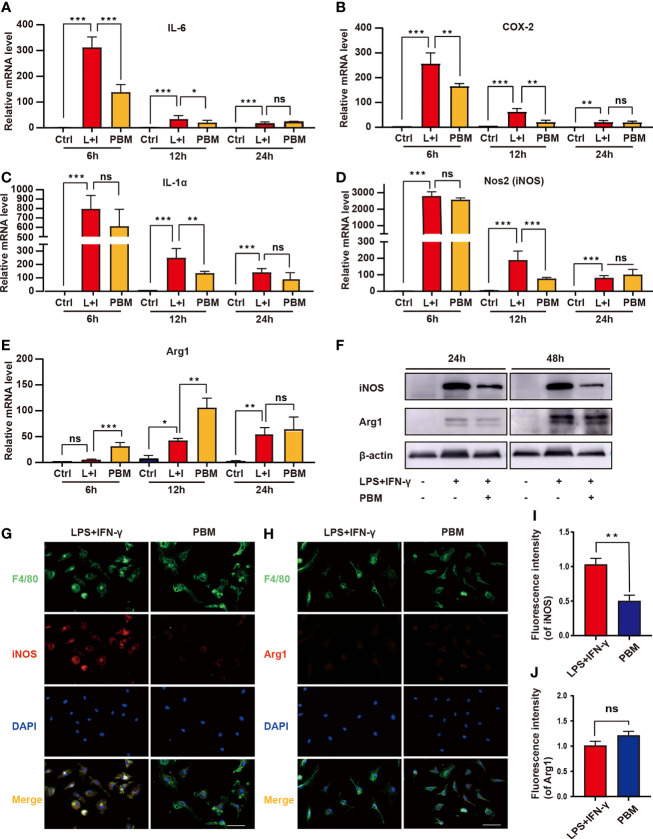
*In vitro* validation of the regulation of inflammation-related genes by PBM. **(A–E)** The expression of M1-related genes (NOS2, IL-1α, IL-6, and COX-2) and M2-related genes (Arg1) in the Ctrl (M0), L+I (LPS + IFN-γ), and PBM (LPS + IFN-γ + PBM) groups was detected by RT–PCR at the 6th, 12th and 24th hours after PBM intervention (n = 3 per group). **(F)** Representative immunoblots of Arg1 (M2 macrophage marker) and iNOS (M1 macrophage marker) in the Ctrl (M0), L+I (LPS + IFN-γ), and PBM (LPS + IFN-γ + PBM) groups at 24 and 48 hours after the first PBM intervention (n = 3 per group). **(G, I)** Representative images of F4/80 (green), iNOS (red) and DAPI (blue) immunofluorescence staining in the LPS + IFN-γ and PBM (LPS + IFN-γ + PBM) groups (n = 3 per group). **(H, J)** Representative images of F4/80 (green), Arg1 (red), and DAPI (blue) immunofluorescence staining in the LPS + IFN-γ and PBM (LPS + IFN-γ + PBM) groups (n = 3 per group at each time point). *P < 0.05, **P < 0.01, ***P < 0.001; ns, not significant.

RNA-seq assays revealed that PBM had the most significant effect on the regulation of inflammatory response-related genes in M1 macrophages (**Figure 4C**). For validation, we selected IL-6, ptgs2 (COX-2), and IL-1α, which had the largest fold difference, as well as iNOS (a marker of M1 macrophages) and Arg1 (a marker of M2 macrophages). Macrophages in the PBM (M1 + PBM) group were treated with PBM every 12 hours, and the expression of IL-6, IL-1α, COX-2, iNOS, and Arg1 was measured at the 6th, 12th, and 24th hours after the first irradiation. Within 12 hours after the first PBM intervention, the expression of IL-6, IL-1α, and COX-2, which is associated with inflammation, decreased to different degrees in the PBM-treated group (**Figures 5A–C**). The time points at which different inflammation-related genes produced significant differences were not identical. The RT–PCR assay of the macrophage markers iNOS and Arg1 revealed that the expression of iNOS decreased and Arg1 increased 12 hours after PBM intervention (**Figures 5D, E**). After PBM intervention, iNOS was significantly reduced in M1 macrophages at the protein level, and no change in Arg1 protein levels was observed (**Figure 5F**). The results of immunofluorescence staining (**Figures 5G–J**) for the macrophage markers iNOS and Arg1 were consistent with the results at the protein level. The above results suggest that PBM exerts anti-inflammatory and pro-damage repair biological effects mainly by inhibiting neurotoxic polarization of M1 macrophages and reducing the secretion of inflammatory factor-associated proteins (**Figure 3F**).

### Effect of PBM on the Notch1-HIF-1α/NF-κB Signalling Pathway in M1 Macrophages

Previous studies have shown that the Notch1 signalling pathway is one of the key pathways regulating the polarization state of macrophages (22, 23). Moreover, Jun Xu et al. (20) found that Notch1 regulates the reprogramming of mitochondrial metabolism in M1 macrophages, demonstrating that NICD1 is recruited to mitochondrial DNA (mtDNA) and that mtDNA-encoded respiratory chain components are upregulated in a Notch-dependent manner. Additionally, the mitochondrial oxidative respiratory chain is an important target that PBM may influence (32). Transcriptome analysis of inflammation-related genes in M1 macrophages showed that PBM significantly downregulated the expression of genes related to classic inflammatory signalling pathways, such as Notch1 and HIF-1α (**Figure 4D**).

**Figure 6:**
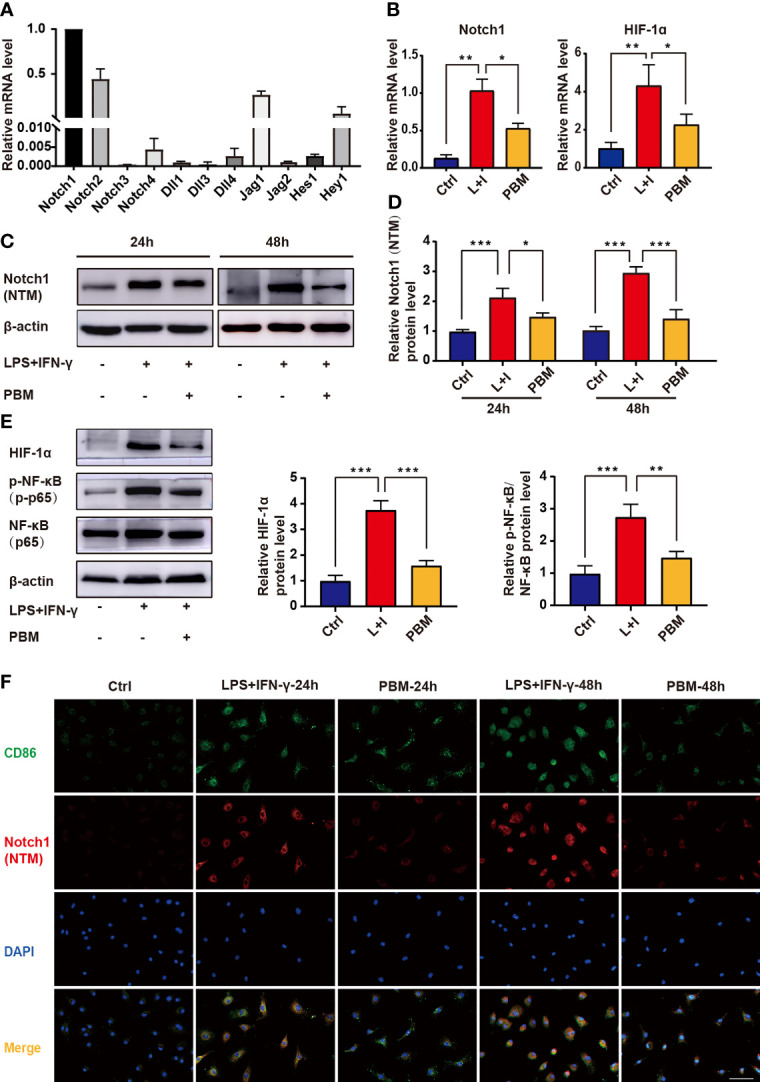
Effect of PBM on the Notch1-HIF-1α/NF-κB signalling pathway in M1 macrophages. **(A)** The mRNA expression levels of Notch signalling pathway-related genes were detected in BMDMs by RT–PCR (n = 3 per group). **(B)** The mRNA expression levels of Notch1 and HIF-1α were detected by RT–PCR in macrophages in the control (Ctrl), L+I (LPS + IFN-γ), and PBM (LPS + IFN-γ + PBM) groups (n = 3 per group). **(C, D)** Protein levels of the cleaved transmembrane/intracellular region of Notch1 (NTM) in the indicated groups were determined by western blotting 24 and 48 hours after the first PBM intervention (n = 3 per group at each time point). **(E)** Protein levels of HIF-1α, NF-κB and p-NF-κB were detected by western blotting of macrophages in the control (Ctrl), L+I (LPS + IFN-γ) and PBM (LPS + IFN-γ + PBM) groups (n = 3 per group). **(F)** Immunofluorescence of Notch-1 (red) and CD86 (M1 macrophage marker; green) in macrophages from different treatment groups (n = 3 per group). *P < 0.05, **P < 0.01, ***P < 0.001.

The RT–PCR results showed that Notch1 was the most highly expressed among the ligand receptors and downstream genes of the Notch signalling pathway in BMDMs (**Figure 6A**). PBM was able to reverse the M1-type polarization of macrophages and elevated Notch1 and HIF-1α gene expression (**Figure 6B**). Assays of protein levels in the cleaved transmembrane/intracellular region of Notch1 (NTM) revealed a significant inhibitory effect of PBM on NTM activation (**Figures 6C, D**). The above results confirm that PBM has a significant inhibitory effect on the activation of Notch1 signalling in M1 macrophages. Assays of the protein levels associated with the Notch1 signalling pathway showed that PBM decreased the protein levels of HIF-1α and p-NF-κB (p-p65) in M1 macrophages (**Figure 6E**). We also observed that PBM had no effect on the protein level of NF-κB in M1 macrophages (**Figure 6E**). To further analyse the effect of PBM on Notch1 expression in M1 macrophages, we used immunofluorescence staining to analyse the expression and localization of Notch1 in macrophages. We observed changes in Notch1 expression between the LPS + IFN-γ and PBM (LPS + IFN-γ + PBM) groups after 24 hours of polarization induction, at which time Notch1 was mainly located at the cell membrane, and Notch1 expression in the PBM intervention group was lower than that in the blank control group (**Figure 6F**). After 48 hours of polarization induction, we also observed a significant decrease in Notch1 entry into the nucleus in the PBM intervention group (**Figure 6F**).

### PBM Regulates M1 Macrophage Polarization *via* the Notch1-HIF-1α/p-NF-κB Axis

**Figure 7:**
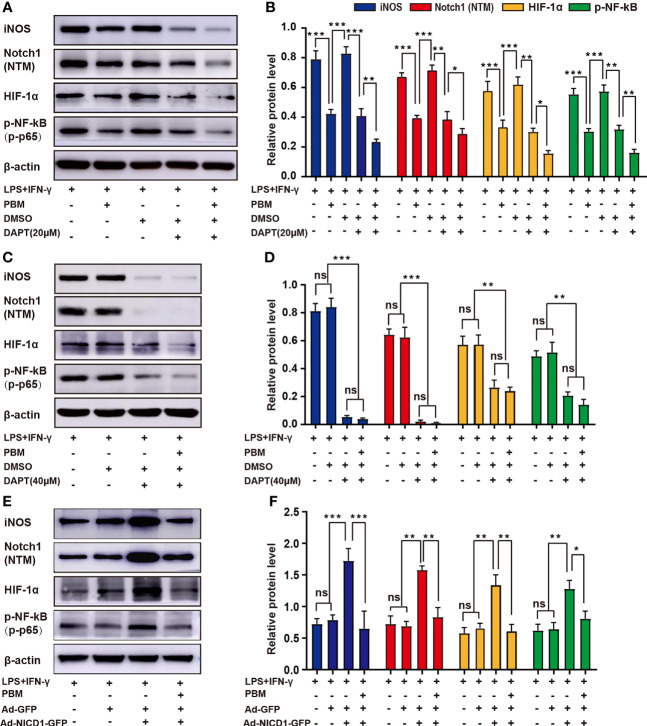
PBM regulates M1 macrophage polarization *via* the Notch1-HIF-1α/p-NF-κB axis. **(A, B)** PBM and DAPT at a concentration of 20 μM synergistically inhibited the neurotoxic polarization of macrophages. Representative blots show the expression levels of iNOS, Notch1 (NTM), HIF-1α, and p-NF-κB in each group. The expression levels of these proteins were also analysed. **(C, D)** DAPT alone at a concentration of 40 μM inhibited Notch-related pathways at low levels, masking the effect of PBM on M1 macrophages. **(E, F)** An adenovirus containing NICD1 was used to activate Notch signalling in macrophages, while the effect of PBM on the protein levels of iNOS, HIF-1α, and p-NF-κB in Notch-activated macrophages was examined by western blotting (all experiments in this group were independently repeated three times). *P < 0.05, **P < 0.01, ***P < 0.001; ns, not significant.

Data from previous experiments have suggested that the Notch1-HIF-1α/p-NF-κB axis may be one of the important pathways through which PBM acts to regulate M1 macrophage-associated inflammatory responses. To further validate the role of the Notch1-HIF-1α/p-NF-κB axis in the regulation of M1 macrophages by PBM, we designed *in vitro* experiments. First, we used DAPT *in vitro* to specifically block the Notch signalling pathway before the induction of macrophage polarization. Similar to the effect of PBM, the application of DAPT alone at a concentration of 20 μM reduced iNOS expression and attenuated the expression of HIF-1α and p-NF-κB. In the control cells, an equal volume of DMSO was added as a vector control for DAPT. DMSO alone did not affect Notch protein expression compared to expression in the no-DMSO control group (**Figures 7A, B**). The combined application of PBM and DAPT enhanced the inhibition of macrophage activation (**Figure 7B**). Application of DAPT alone at a concentration of 40 μM directly inhibited Notch signalling to very low levels, thus masking the regulatory effect of PBM on Notch (**Figures 7C, D**). Next, we altered the level of NICD1 in macrophages by adenoviral transfection. The results showed that adenoviral transfection effectively elevated Notch1 (NTM) levels while leading to activation of HIF-1α/p-NF-κB in neurotoxic macrophages (**Figures 7E, F**). Combined with NICD1-overexpressing adenovirus and PBM, PBM reversed the activation of Notch1, HIF-1α, and p-NF-κB to some extent (**Figure 7F**).

## Discussion

The pathological process of SCI is complex and can be divided into two main stages: primary and secondary injury (1, 3, 5). Primary injuries are mostly caused by physical factors and are irreversible. Therefore, secondary injuries have become the focus of attention. Secondary injury mainly includes the inflammatory response, local ischaemia and apoptotic necrosis (7). Promoting neuronal repair and recovery of motor function by modulating the secondary inflammatory response is one of the key strategies in the treatment of SCI (6). Macrophages are one of the most important cells in the inflammatory response of SCI (6, 8, 13) and can be polarized into proinflammatory M1 macrophages or anti-inflammatory M2 macrophages; their polarization status plays a decisive role in the regulation of the inflammatory microenvironment (33). Although macrophage polarization can be regulated to some extent by genetic engineering techniques and cell transplantation (34–37), there are immune rejection reactions, tumorigenic risks, and ethical concerns. Finding a safe and effective method to modulate the secondary inflammatory response in the SCI area has become urgent.

Our group has been investigating the potential of PBM in the treatment of SCI (15, 30). Related studies found that direct percutaneous irradiation of the SCI area in rats with 810-nm NIR light promoted the activation of anti-inflammatory M2 macrophages and the survival of neurons in the injury area (15, 16). However, percutaneous irradiation methods inevitably result in a loss of light energy, making accurate localization of the injury and assessment of the energy in the injury area extremely difficult, which greatly increases the uncertainty of the treatment outcome. Especially for deep spinal cord tissues, accurate assessment of the effective irradiation dose to the injury area is critical to the success of PBM treatment. Studies related to PBM treatment in rat SCI models have shown that the infrared transmission rate of percutaneous irradiation for SCI is only 6% (38). The low transmission rate severely limits the practical application of PBM therapy in SCI.

In this study, we used an implantable biofibre to deliver 808-nm NIR light directly to the surface of the injured spinal cord (25, 26). This PBM treatment model inhibited neurotoxic polarization of macrophages and secretion of inflammatory factors in the SCI area of mice, leading to a significant enhancement of motor function. In contrast, there was no significant effect on the expression of anti-inflammatory M2 macrophages in the injury area. Our study also found that PBM reduced the apoptosis rate of VSC 4.1 motoneurons and promoted axonal extension of DRG neurons. This is somewhat different from previous studies on the regulation of inflammatory responses by PBM in spinal cord-injured rats. Previous findings (15, 16) suggested that PBM significantly elevated the proportion of anti-inflammatory M2 macrophages in the injury area. This discrepancy may be related to the choice of experimental animals and models of PBM intervention. Our experimental results suggest that M1 macrophages may be one of the main targets by which PBM exerts its anti-inflammatory effects. RNA-seq analysis was also used to study the biological effects of PBM on M1 macrophages and to identify the main DEGs. The GO analysis results showed that PBM inhibited the expression of inflammation-related genes in neurotoxic macrophages. We screened for five inflammation-related genes to examine changes in the regulatory effect of PBM based on the time of testing and the irradiation dose (irradiation given every 12 hours). A clear biphasic dose–response curve was found for the regulation of inflammatory gene expression by PBM at both the gene and protein levels. That is, when the irradiation dose was too low or too high (fluence (J/cm^2^), irradiance (mW/cm^2^), irradiation time, or the number of irradiations), PBM did not have apparent effect. This biphasic response follows the pattern of a weak stimulus producing a weak effect, a strong stimulus further increasing the corresponding biological effect until it reaches a peak, and an even stronger stimulus causing the biological effect to disappear or even produce the opposite effect. The irradiation dose thresholds corresponding to the most significant effects vary slightly from gene to gene. However, for the five inflammation-related genes we investigated, there were significant peaks in the modulatory effects of PBM with the number of irradiations and the time point of detection. Overall, 12 hours after PBM stimulation, the most significant inhibitory effect of PBM on inflammatory genes was observed; by 48 hours, this modulatory effect was diminished. Similar results were found at the protein level. Our findings demonstrate that PBM has a biphasic dose–response curve (39, 40).

To investigate the molecular mechanisms by which PBM regulates the inflammatory response of M1 macrophages, we analysed DEGs associated with the regulation of macrophage inflammatory polarization and found that PBM downregulated the expression of the Notch1 and HIF-1α genes while exerting an anti-inflammatory effect. Our studies on mouse BMDMs revealed that LPS plus IFN-γ promoted the expression of Notch1, HIF-1α and p-NF-κB and induced the polarization of BMDMs towards M1 macrophages. In contrast, after treating LPS plus IFN-γ-induced M1 macrophages with DAPT, an inhibitor of the Notch pathway, the inhibition of the Notch signalling pathway was accompanied by a significant decrease in HIF-1α and p-NF-κB protein levels. Overexpression of NICD1 in mouse bone marrow-derived M1 macrophages was accompanied by increased expression of HIF-1α and p-NF-κB. The above findings suggest that in LPS plus IFN-γ-induced M1 macrophages, Notch1 activation is necessary for inflammatory activation of macrophages and that HIF-1α and p-NF-κB activation is largely dependent on the Notch1 pathway. The Notch1-HIF-1α/p-NF-κB signalling pathway axis thus plays an important role in LPS- and IFN-γ-induced M1-type activity in mouse BMDMs. Previous studies have shown that LPS-activated M1 macrophages exhibit activation of the Notch1 (22), HIF-1α (21, 41) and NF-κB signalling pathways (23). The activation of all three signalling pathways promotes the neurotoxic polarization of macrophages and the secretion of corresponding inflammatory factors (21–23, 41). In contrast, a study on mouse liver macrophages and RAW264.7 cells showed that a significant portion of the activation of HIF-1α and p-NF-κB by LPS was achieved through Notch1. Knockdown of Notch1 in macrophages resulted in significantly diminished activation of HIF-1α and p-NF-κB by LPS and significantly reduced expression of the M1 macrophage marker iNOS (20). This finding is similar to our conclusion; that is, whether in mouse bone marrow-derived M1 macrophages induced by LPS plus IFN-γ or in mouse liver macrophages and RAW264.7 cells induced by LPS alone, the Notch1-HIF-1α/p-NF-κB axis plays an important role in regulating the inflammatory polarization of macrophages, and Notch1 is at the core of the pathway. We also confirmed that the regulation of neurotoxic macrophage inflammation by PBM is achieved, at least in part, through the Notch1-HIF-1α/p-NF-κB signalling pathway axis. PBM was found to play a role similar to that of Notch pathway inhibitors. Inhibition of Notch1 expression in M1 macrophages was accompanied by the decrease of HIF-1α and p-NF-κB expression and the disturbance of neurotoxic activation of macrophages. Moreover, PBM reversed the overexpression of NICD1 in M1 macrophages to some extent.

**Figure 8:**
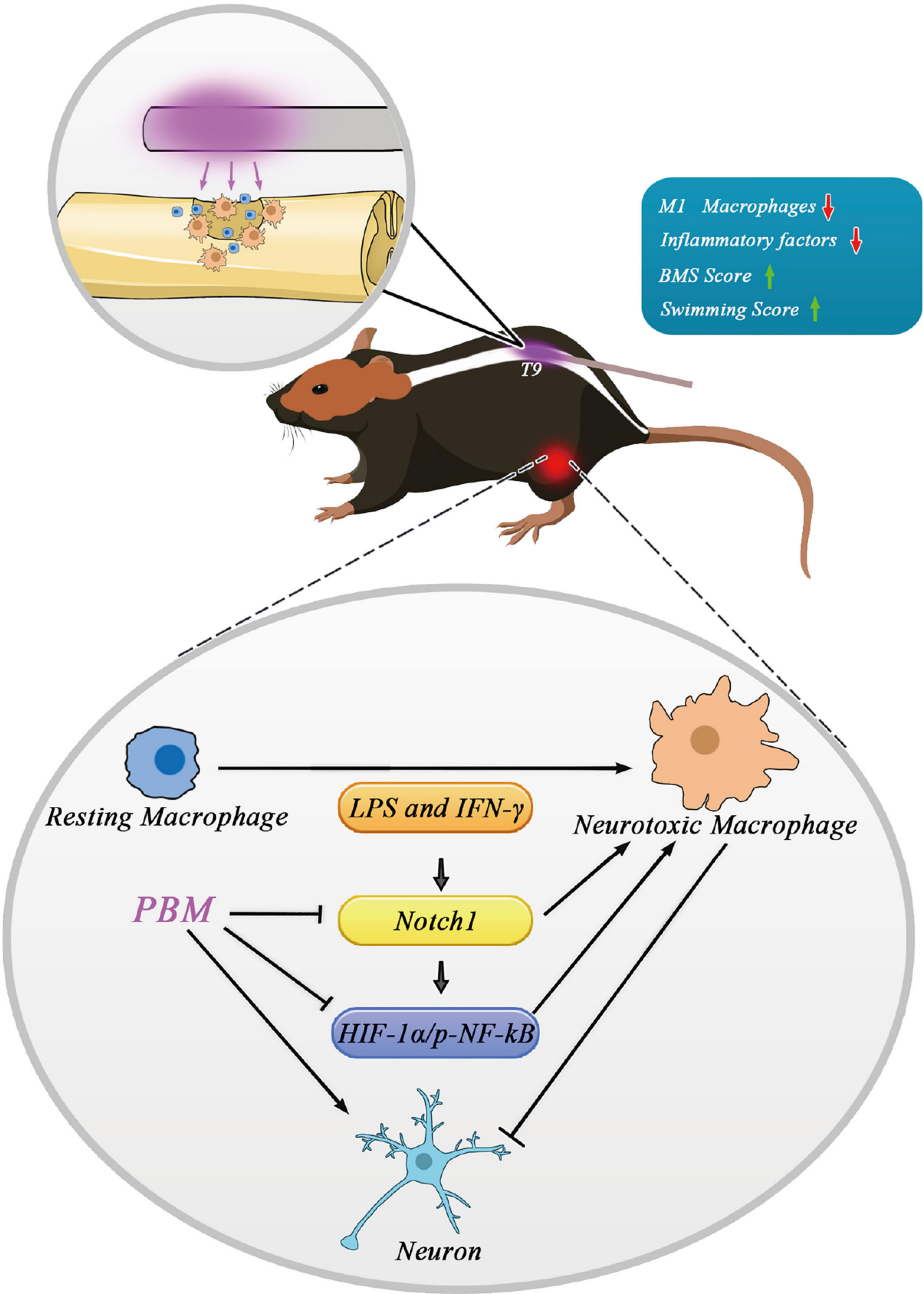
Schematic model showing the regulatory role of PBM on M1 macrophages and its possible molecular mechanisms. Macrophages in the resting state become activated and are involved in neuroinflammation after spinal cord injury. Activated neurotoxic macrophages have damaging effects on neurons by secreting cytotoxic cytokines. The Notch1 and HIF-1α/p-NF-κB axis are involved in macrophage activation. Specifically, the Notch1 pathway is involved in the activation of neurotoxic macrophages and the expression of HIF-1α and p-NF-κB. The neuroprotective mechanism of PBM may be related to its inhibition of the Notch1-HIF-1α/p-NF-κB axis, which is associated with the activation of neurotoxic macrophage cells.

In summary, as shown in the schematic diagram (**Figure 8**), direct irradiation of the spinal cord injury area with *in vivo* buried biomedical optical fibres can promote the recovery of motor function in the lower limbs of SCI mice by inhibiting neurotoxic macrophage activation and reducing cytotoxic cytokine secretion. The potential mechanisms of action may be multifaceted. For example, PBM inhibited the neurotoxic polarization of macrophages through modulation of their polarization-related Notch1-HIF-1α/p-NF-κB axis while reducing the expression of inflammation-related IL-6, IL-1α, and COX-2. The reduction in neurotoxic cytokine secretion indirectly promoted neuronal survival and the recovery of motor function. Our findings provide new insight into the biological role, regulatory targets and molecular mechanisms of PBM in spinal cord injury. The main shortcomings of this study are as follows. First, the examination of signalling pathways related to PBM was limited to *in vitro* experiments and lacked validation of the related pathways *in vivo*. In response to this problem, our group will further construct myeloid-specific Notch1 knockout mice, and on this basis, study the role of Notch1 signalling pathway in the regulation of macrophage polarization in the spinal cord injury area by PBM. Second, we focused only on the effect of PBM on the inflammatory response associated with macrophage polarization in the early stages of SCI, while the targets and biological functions of PBM may be diverse. Additionally, we used only male mice in this study, and the relevant findings thus cannot be extrapolated to female mice or other animals or injury models without further experimental investigations. Therefore, in the next step, we will design corresponding experiments to fully elucidate the mechanisms and possible targets of PBM to promote recovery from SCI and address these shortcomings.

## Data Availability Statement

The datasets presented in this study can be found in online repositories. The names of the repositories and accession numbers can be found below: https://www.ncbi.nlm.nih.gov/geo/, GSE188917.

## Ethics Statement

The animal study was reviewed and approved by The Ethics Committee of the Fourth Military Medical University.

## Author Contributions

YM, PL, and CJ contributed equally to this work and share the first authorship. YM, TD, XH, and ZW designed and conceived the study. PL, CJ, and XL built the animal model and conducted PBM intervention. XZ, ZL, JZ, KL, XW, ZJZ, ZS, and ZHZ performed the experiments. YM, CJ, and XW contributed to the analysis. The manuscript was written by YM, PL, and CJ. XH and ZW critically contributed to and revised the manuscript. All authors contributed to the article and approved the submitted version.

## Funding

National Natural Science Foundation of China (81070996, 81572151); Shaanxi Provincial Science and Technology Department (2021SF-029, 2021ZDLSF02-10, 2020ZDLSF02-05); The Everest Project of Fourth Military Medical University (2018RCFC02).

## Conflict of Interest

The authors declare that the research was conducted in the absence of any commercial or financial relationships that could be construed as a potential conflict of interest.

## Publisher’s Note

All claims expressed in this article are solely those of the authors and do not necessarily represent those of their affiliated organizations, or those of the publisher, the editors and the reviewers. Any product that may be evaluated in this article, or claim that may be made by its manufacturer, is not guaranteed or endorsed by the publisher.
